# Cannabidiol and Alzheimer Disease: A Comprehensive Review and In Silico Insights Into Molecular Interactions

**DOI:** 10.1111/ejn.70229

**Published:** 2025-08-27

**Authors:** João V. Mello‐Hortega, Carolina S. de Oliveira, Vitoria S. de Araujo, Lupe Furtado‐Alle, Luciane V. Tureck, Ricardo L. R. Souza

**Affiliations:** ^1^ Polymorphisms and Linkage Laboratory, Postgraduate Program in Genetics, Department of Genetics Federal University of Paraná (UFPR) Curitiba Paraná Brazil

**Keywords:** Cannabinoids, Dementia, Enrichment Pathways, Neuroprotection

## Abstract

Alzheimer's disease (ad) is a neurodegenerative disorder characterized by a set of multifactorial conditions that progressively impair memory processing and cognitive function. The study of this pathology is particularly challenging due to its complex etiology, which involves several pathological hallmarks, including amyloid plaque formation, tau protein hyperphosphorylation, neuroinflammation, oxidative stress, and other contributing factors—all leading to neuronal loss. The primary therapeutic approach for AD involves the use of anticholinesterase agents; however, these treatments are associated with adverse effects, and their efficacy has been increasingly questioned. Against this backdrop, researchers have investigated cannabidiol (CBD) as a potential complementary treatment for AD. This study compiles and synthesizes current evidence regarding the therapeutic effects of CBD in the context of AD, examining its impact on the amyloid cascade, tau phosphorylation, neuroinflammation, oxidative stress, the cholinergic pathway, glucose and lipid metabolism, behavioral alterations, and physiological changes. In addition, an in silico analysis was conducted based on studies that identified differential gene expression in response to CBD. Through this analysis, we mapped the gene network and biological pathways involved in CBD's mechanism of action in ad, contributing to the identification of potential gene targets for further research and providing deeper insight into its therapeutic potential.

## Graphical abstract

Cannabidiol improves several Alzheimer's‐related aspects and regulates disease‐associated genes. Literature review and Over‐Representation Analysis of 64 genes confirm its effects on key mechanisms such as amyloid‐β and tau processing, oxidative stress, inflammation, and lipid metabolism. These findings highlight signaling pathways as targets and support CBD's therapeutic potential in Alzheimer's disease.

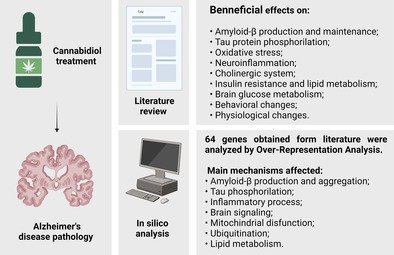

**Abbreviations**
Aβ: amyloid‐β
AChE: acetylcholinesterase
AD: Alzheimer's disease
AKT: protein kinase 3
APP: amyloid precursor protein
ARRB2: beta‐arrestin‐2
BACH1: BTB and CNC homology 1
BChE: butyrylcholinesterase
BPSD: behavioral and physiological symptoms of dementia
CB1: cannabinoid receptor 1
CBD: cannabidiol
CDK: cyclin‐dependent kinase
CNPq: Conselho Nacional de Desenvolvimento Científico e Tecnológico
CNS: central nervous system
DNM1L: hippocampal dynamin 1 like
ERKs: extracellular signal‐regulated kinases
FAAH: fatty acid amide hydrolase
GFAP: glial fibrillary acidic protein
GSK3: glycogen synthase kinase 3
HMOX1: heme oxygenase 1
HSPs: heat shock proteins
iNOS: inducible nitric oxide synthase
LTP: long‐term potentiation
MAPK: mitogen‐activated protein kinase
NRF2: nuclear factor erythroid 2–related factor 2
ORA: over‐representation analysis
PET: positron emission tomography
PPAR‐γ: peroxisome proliferator‐activated receptor gamma
RNS: reactive nitrogen species
ROS: reactive oxygen species
TREM2: triggering receptor expressed on myeloid cells 2
TRPV2: transient receptor potential cation channel subfamily V member 2

## 1 Introduction

With the increase in life expectancy worldwide aided by modern medicine, dementia has emerged as a global health challenge. In 2022, it is estimated that 55.2 million individuals will be affected globally, with 60% to 80% of cases triggered by Alzheimer's disease (ad) (Prince 2015; “2022 Alzheimer's disease facts and figures” 2022).

ad is a neurodegenerative disease that results in a set of disorders that progressively affect functions related to memory capacity, clear reasoning, and can affect the functional performance of activities considered complex (Corey‐Bloom et al. 1995; Abreu et al. 2005; Williams et al. 2010).

The development of ad can be influenced by factors such as genetic variants, aging, smoking, vascular problems, obesity, among others (Silva et al. 2019; Zhang et al. 2021). The genetic factors associated with the disease include variants in the amyloid precursor protein (gene *APP*) and the presenilin 1 and 2 genes (*PSEN1* and *PSEN2*), which are commonly linked to early‐onset or familial ad, while late‐onset ad is primarily associated with variants in the gene encoding apolipoprotein E (*APOE*) (Tanzi 2012; Pereira et al. 2021).

In addition to the factors that may influence the development of ad, different pathological pathways are observed as the disease progresses, contributing to the complexity associated with this condition. Despite efforts to understand it, studies have identified various pathological hallmarks that lead to neuronal loss. Among them, the amyloid‐β (Aβ) and tau protein pathways are the most described and well established in association with ad. In the ad brain, the exacerbated expression of Aβ proteins results in accumulation between neurons, challenging the proper functioning of synapses and resulting in cognitive deficits. In addition to the formation of Aβ plaques, there are also neurofibrillary tangles, caused by abnormal expression and tau protein hyperphosphorylation (Reitz et al. 2011). This protein plays an important role in maintaining neuronal structure and, when abnormal, clusters in a disorganized way inside cells, disrupting tissue homeostasis. These processes are the most commonly AD pathological hallmarks, being one of the main diagnostic criteria for the pathology (Mariani 2004).

Neuroinflammation and oxidative stress are other processes that appear to mediate the development of AD, resulting in the activation of microglia and astroglia as a form of defense of brain tissue. These mechanisms are mediated by the release of pro‐inflammatory cytokines and the excessive production of reactive oxygen species (ROS) that can cause neuronal death and the consequent cognitive deficit observed in AD (Klegeris et al. 1994; Meda et al. 1995; Hashioka et al. 2021). In addition to these mechanisms, other hypotheses have contributed to a better understanding of the pathology. Notable among them are the metal ion hypothesis, the abnormal autophagy hypothesis, the microbiota–gut–brain axis hypothesis, the glutamate excitotoxicity hypothesis, and the cholinergic hypothesis (Zhang et al. 2024).

The cholinergic hypothesis plays a relevant role in the treatment of patients, since neurochemical changes are observed in brains diagnosed with ad, such as a decrease in the enzyme responsible for the production of acetylcholine in brains with the presence of neuritic plaques (Perry et al. 1978; Cummings and Back 1998; Hampel et al. 2018). Therefore, currently one of the only palliative treatments for ad consists of cholinesterase inhibitors use, which act on this system by blocking the cholinesterases, responsible for metabolizing acetylcholine, aiming to increase neurotransmitter levels in synaptic clefts, thus enabling better communication between neurons (Weinreb et al. 2009). However, this method presents undesirable side effects, such as nausea, circulatory problems, dizziness, insomnia, among others, and its effectiveness has been questioned in some studies, highlighting the need for the development of alternative therapeutic approaches (Hampel et al. 2018; Han et al. 2019; Xu et al. 2021).

Considering the need for the development of new therapeutic approaches, many substances have already been tested in pre‐clinical and clinical studies for ad treatment (Li et al. 2023; Smith and Ownby 2024). Among these substances, cannabidiol (CBD) stands out, as it is a common cannabinoid that does not produce psychodysleptic effects and exhibits anti‐inflammatory and antioxidant properties, among other effects (Hampson et al. 1998; Woelfl et al. 2020). Due to its therapeutic potential, many studies have sought to evaluate the effects of CBD in the context of AD, as well as to identify the pathways through which this cannabinoid exerts its action.

In this context, the aim of the present study was to obtain a comprehensive understanding of CBD therapy in ad and to identify the main biological pathways affected by treatment with this cannabinoid, through an in silico analysis based on genes modulated by CBD. Thus, we sought to gather information on the potential therapeutic applications of CBD in ad and to provide insights into the molecular interactions underlying its effects on the disease.

## 2 Methodology

To provide a comprehensive overview of CBD therapeutics, a narrative review was conducted based on searches for scientific papers in the PubMed and Web of Science databases, using the keywords “Cannabidiol” and “Alzheimer's.” At this stage, no filters were applied regarding the year of publication or article type.

The initial search retrieved 144 papers, which were subsequently screened to exclude review articles or those that only mentioned the search terms without directly addressing the topic. After this filtering process, 45 original research articles were selected to examine the effects of CBD on the pathogenesis of ad (Supplementary Table S1). These studies encompass a wide range of evidence from in vivo, in vitro, and in silico models, highlighting various biological pathways modulated by CBD. An in silico analysis was conducted with the aim of mapping the interactions between genes and CBD and subsequently identifying the biological pathways modulated by the gene products affected by this compound. For this purpose, we selected from the 45 reviewed studies those that evaluated gene expression in response to CBD treatment using RNA‐seq or microarray technologies. From this selection, two articles (Aso et al. 2014; Libro et al. 2016) were used to generate a gene set consisting of 64 genes modulated by CBD treatment (see Supplementary Table S2). This list includes all differentially expressed genes listed in these works related to ad. These studies were chosen for pathway enrichment analysis because they provided gene lists associated with ad and modulated by CBD treatment, obtained through hypothesis‐free analytical methods. This approach reduces bias and increases the reliability of identifying relevant biological pathways.

An Over‐Representation Analysis (ORA) was then performed using the clusterProfiler package (Yu et al. 2012) in R, with the KEGG gene set database. This technique evaluates whether specific biological pathways or processes are over‐represented or enriched in a list of experimentally identified genes compared to what would be expected by random chance. The parameters set were as follows: pvalueCutoff = 0.05, qvalueCutoff = 0.05, universe = org.Hs.eg.db, minGSSize = 10, maxGSSize = 500.

## 3 Results and Discussion

### 3.1 CBD and AD Pathways

**FIGURE 1:**
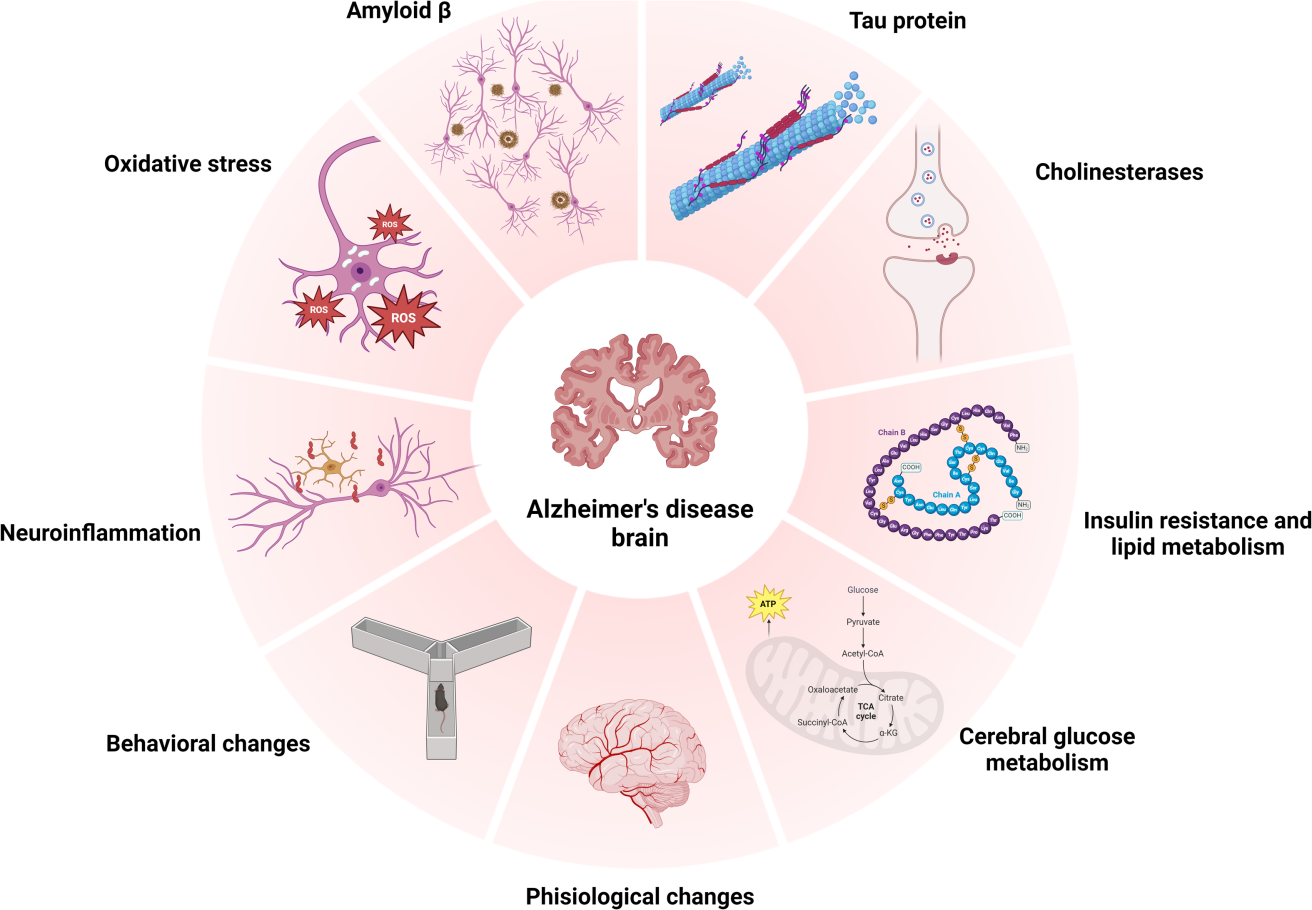
Alzheimer's disease (ad) factors affected by cannabidiol (CBD) treatment: production and maintenance of beta amyloid, phosphorylation of tau protein, oxidative stress, neuroinflammation, cholinergic system, insulin resistance and lipid metabolism, brain glucose metabolism, behavioral changes, and physiological changes.

Findings in the literature demonstrate the beneficial effects of CBD for the treatment of ad. This evidence was grouped into nine categories that contemplate the effects of this cannabinoid reported in the literature, represented in Figure 1.

**FIGURE 2:**
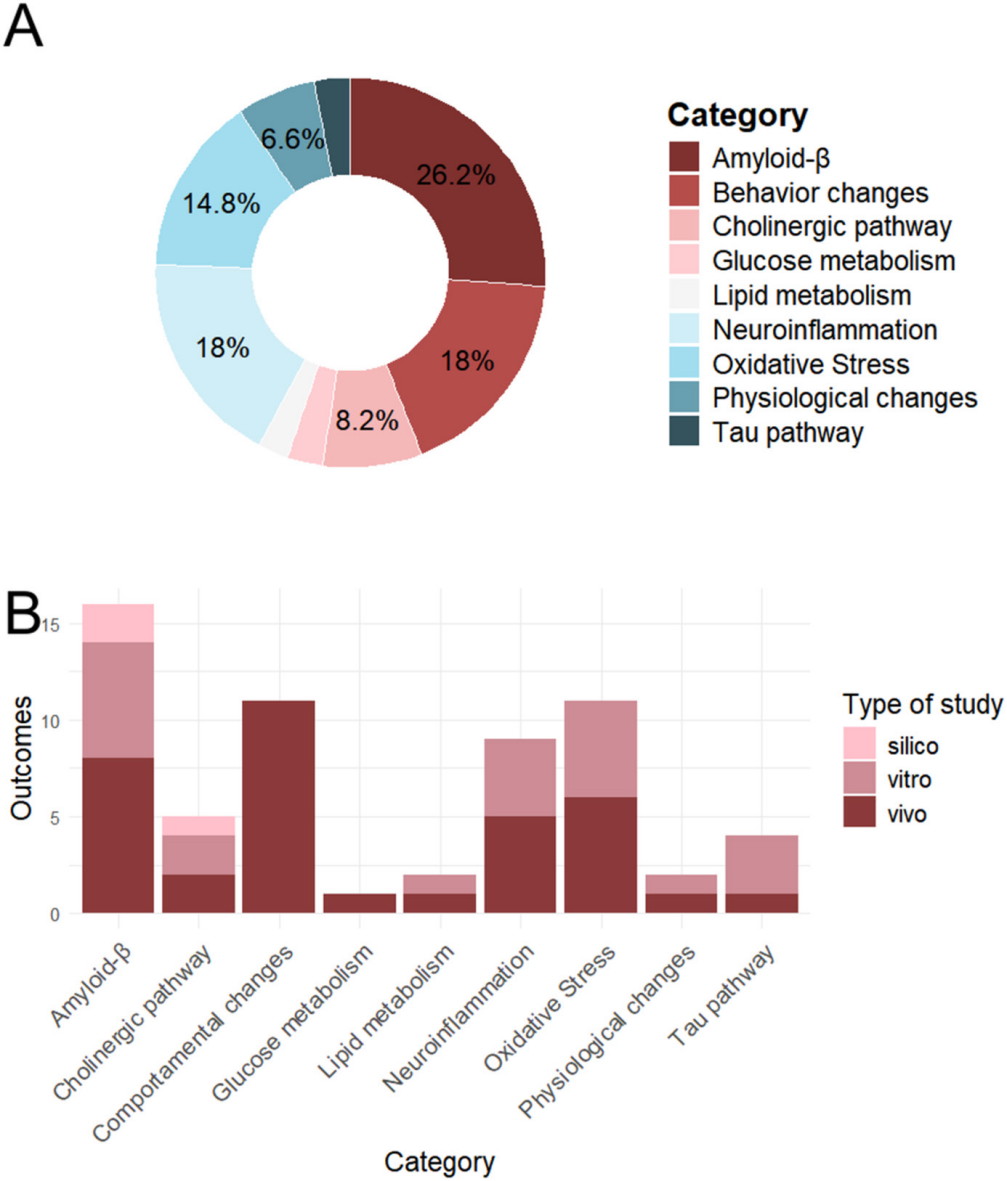
Proportion of outcomes of the effects of CBD in AD therapy separated by categories and type of study. The analysis of the 45 papers found in the literature made it possible to gather a set of 61 outcomes that experimentally demonstrate the beneficial effect of CBD treatment in AD therapy. (a) Proportion of outcomes of the effects of CBD in AD therapy separated by categories, such as: production and maintenance of Aβ, phosphorylation of tau protein, oxidative stress, neuroinflammation, cholinergic system, insulin resistance and lipid metabolism, brain glucose metabolism, behavioral changes, and physiological changes. Only percentages > 5% are shown in the figure. (b) Proportion of study type by category, separated by in vivo, in vitro and in silico studies.

Among the 45 articles analyzed, different study methods were applied in order to better understand the effects of CBD on ad. In these works, outcomes were identified that demonstrate the beneficial effect of CBD in the treatment of ad. Studies involving analysis of mechanisms of the amyloid pathway, oxidative stress, and neuroinflammation were the most abundant, as well as behavioral analysis in in vivo models treated with CBD. The proportion of outcomes related to each category of the disease addressed can be seen in Figure 2, as well as the proportion of the type of analysis used for these inferences.

#### 3.1.1 CBD in Aβ Production, Aggregation, and Immune Response

The Aβ pathway is well recognized in ad and most studies have evaluated CBD effects on the production and accumulation of the proteins and the immune response linked to the presence of these aggregates.

A study using mesenchymal stem cells found, after the treatment with CBD, lower expression of genes encoding for secretases, enzymes responsible for cleaving the APP, resulting in the formation of smaller Aβ fragments (De Strooper et al. 2010; Libro et al. 2016). CBD was described as a potential inhibitor of the β‐secretase activity (Vallée et al. 2017). In relation to other mechanisms of Aβ production, the regulation of the Wnt/β‐catenin pathway and Peroxisome Proliferator‐Activated Receptor Gamma (PPAR‐γ) also appear to be affected by CBD treatment, resulting in decreased production of the Aβ precursor; besides that, CBD acts as an inverse agonist, inhibiting the beta‐arrestin‐2 (ARRB2) recruitment and also in the ubiquitination of APP, decreasing Aβ production (Scuderi et al. 2014; Vallée et al. 2017; Woelfl et al. 2020).

In addition to acting on the Aβ production, CBD has an important effect on the accumulation of these proteins, which form the protein aggregates. Several studies using in vivo models have demonstrated the role of CBD in reducing processes related to the Aβ accumulation, such as decreasing levels of soluble Aβ and reduced deposition in the hippocampus and cerebral cortex (Casarejos et al. 2013; Aso et al. 2014; Watt et al. 2020; Amini and Abdolmaleki 2022; Zhang et al. 2022). An in silico study reinforces the idea of CBD's regulatory role in reducing the aggregation of these peptides at certain concentrations of use (Chrobak et al. 2021). One of the hypotheses for the reduction in protein aggregation is related to CBD's ability to scavenge ROS. This process may be related to the protective function of the Aβ protein, so that, with the reduction in stress caused by ROS, the signaling for this protein production is reduced (Zhang et al. 2022).

Still in relation to these aggregates, but now considering the effects associated with its presence, the major impact is on neuronal functioning; but also, immune response aiming at re‐establishing tissue homeostasis is closely associated. In this context, microglia play an important role in the response to ad pathology. Microglia comprise a group of cells in the central nervous system responsible for the maintenance and defense of neurons. Activation of these cells is commonly due to infections, injuries, and neurodegenerative diseases (Prinz et al. 2019; Cserép et al. 2020). According to the functions performed, microglia play an important role in combating the protein aggregates. Studies have shown that CBD decreases the expression of genes related to specific actions of microglia. These actions are related to the promotion of an immune response through pathways related to autophagy‐related process and phagocytosis, which occur through the activation of the transient receptor potential cation channel subfamily V member 2 (TRPV2), in order to reduce the neurotoxicity promoted by Aβ (Hao and Feng 2021; Yang et al. 2022; Duan et al. 2023; Marsh et al. 2023).

#### 3.1.2 CBD Modulates Tau Protein Aggregation and Phosphorylation

The tau protein pathway is a well‐established ad pathway. Studies have already shown that CBD has a beneficial effect in reducing tau aggregation, since this compound spontaneously binds to the protein, hindering the process of protein accumulation and deposition in the cortex and hippocampus (Casarejos et al. 2013; Alali et al. 2021). Regarding protein phosphorylation, in vitro studies have shown the influence of CBD on the downregulation of genes encoding kinases, enzymes responsible for the phosphorylation of various molecules, including the tau protein. An important kinase in the tau protein phosphorylation pathway is glycogen synthase kinase 3 (GSK3), which phosphorylates tau on most of the hyperphosphorylated serine and threonine residues in paired helical filaments; GSK3 activity contributes to both Aβ production and Aβ‐mediated neuronal death. This enzyme is regulated by several mechanisms, including the Wnt signaling pathway mediated by β‐catenin, which explains other results found in studies that indicate a regulatory role of CBD in this signaling pathway, decreasing the phosphorylation of the tau protein (Esposito, De Filippis, Carnuccio, et al. 2006; Wu and Pan 2010; Hernandez et al. 2012; Aso et al. 2014; Libro et al. 2016).

#### 3.1.3 CBD as a Modulator of Neuroinflammation

Neuroinflammation is another important mechanism related to the ad pathogenesis, especially in the development and worsening of associated dementia (Zhang et al. 2013). Given the CBD mechanisms, several researchers have made efforts to verify inflammation‐related therapeutic effects in models of the disease (Esposito, De Filippis, Carnuccio, et al. 2006; Esposito et al. 2007, 2011; Martín‐Moreno et al. 2011; Khodadadi et al. 2021; Wang et al. 2021; Landucci et al. 2022). Some associations have been identified between CBD treatments and the activation of microglia, a process in which these cells switch from their “resting” state (due to disturbances in brain homeostasis) to an active state to counteract these disturbing factors (Kettenmann et al. 2011).

Activated microglia, regarding in vitro models, can be classified in two ways: the M1 phenotype presenting pro‐inflammatory and neurotoxic activity; the M2 phenotype plays a role in attenuating the inflammatory process by releasing anti‐inflammatory cytokines and growth factors, eliminating debris by inducing phagocytic activity, and remodeling the tissue (Cherry et al. 2014; Hu et al. 2015; Orihuela et al. 2016; Simon et al. 2017). An in vitro study demonstrates the effect of CBD in preventing the transition of microglia from the M0 (resting) stage to the M1 stage, blocking the activation of these cells and reducing the pro‐inflammatory effect on the disease (Landucci et al. 2022).

CBD acts on decreasing the release of cytokines (such as proinflammatory interleukins) and other proteins such as inducible nitric oxide synthetase (iNOS). The latter releases nitric oxide in certain stress conditions, which can result in the release of reactive nitrogen species, causing further damage to the tissue (Esposito, De Filippis, Maiuri, et al. 2006; Esposito et al. 2007; Martín‐Moreno et al. 2011; Khodadadi et al. 2021). In addition to iNOS, glial fibrillary acidic protein (GFAP) also had its expression decreased with CBD treatment (Esposito et al. 2007). In a study that evaluated neuroinflammation and the expression of this protein in rats subjected to a hypercaloric diet, an exacerbated GFAP expression was related to the impairment in short‐ and long‐term memory (Bondan et al. 2019). This indicates a possible relationship between the expression of GFAP and the deficiency in memory processing observed in ad.

Corroborating the anti‐inflammatory effects through the molecules expression inhibition, in studies based on the *Caenorhabditis elegans* ad model, CBD promoted greater expression of the gene encoding the triggering receptor expressed on myeloid cells 2 (TREM2); in order to increase the phagocytic response in the tissue, as well as increasing the expression of fatty acid amide hydrolase (FAAH) and the cannabinoid receptor 1 (CB1) (Esposito et al. 2007; Khodadadi et al. 2021; Wang et al. 2021).

In addition to activating and modulating microglia, CBD also has an anti‐inflammatory effect through the PPAR‐γ receptor. This receptor is overexpressed in patients diagnosed with ad and acts in the regulation of lipid and insulin metabolisms, demonstrating anti‐inflammatory effects in other types of central nervous system (CNS) diseases (Landreth et al. 2008). (Landreth et al. 2008). An in vivo ad study model showed that CBD acted as an agonist of the PPAR‐γ receptor, reducing the inflammatory state, consequent neuronal damage, and has been shown to promote hippocampal neurogenesis (Esposito et al. 2011).

#### 3.1.4 Antioxidant Effects of CBD and Modulation of Oxidative Stress

Like neuroinflammation, oxidative stress has been increasingly studied in the context of ad, and it consists of dysregulation in the production of ROS, reactive nitrogen species (RNS), and antioxidant defense mechanisms. Due to this imbalance, these unstable and reactive molecules can transform other molecules into which they collide, causing detrimental effects (Halliwell and Gutteridge 2015). This damage has been linked to the worsening of ad‐related dementia, making oxidative stress‐promoting pathways a promising target for combating the progression of the disease (Halliwell and Gutteridge 2015).

Some studies have already shown a positive effect of CBD in regulating pathways related to the production of free radicals, reducing ROS without inducing the overexpression of oxidative genes (Iuvone et al. 2004; Esposito et al. 2011; Casarejos et al. 2013; Scuderi et al. 2014; Frandsen and Narayanasamy 2022; Vanin et al. 2022; Wang et al. 2023). In a *C. elegans* model, a preventive CBD effect on cell damage caused by methylglyoxal, a reactive compound resulting from the glyoxalase neural pathway, was found (Frandsen and Narayanasamy 2022). In PC12 and SH‐SY5Y cell lines, a beneficial nature of CBD was observed in relation to cell viability in response to Tert‐Butyl Hydroperoxide, an oxidizing compound (Harvey et al. 2012). CBD also has a role in reducing oxidative stress related to the dopamine metabolism pathway, the Wnt/β‐catenin pathway, and the PPAR‐γ receptor (Scuderi et al. 2014; Vallée et al. 2017). Furthermore, studies using a CBD derivative report its action on the axis of the transcription factor NF‐E2‐related factor 2 (NRF2) and the transcription factor BTB and CNC homology 1 (BACH1). This derivative acts by inhibiting the repressive action of BACH1 in the promoter region of the gene responsive to oxidative stress, heme oxygenase 1 (HMOX1), conferring an antioxidant and cytoprotective effect on cells (Casares et al. 2020).

The antioxidant role of CBD, in addition to acting directly on pathways that produce reactive molecules, also plays an important part in modulating genes related to mitochondrial dynamics (Iuvone et al. 2004; Harvey et al. 2012; da Silva et al. 2014; Rajan et al. 2017). This cannabinoid was able to rescue effects caused by the induction of damage using iron in a model of neurodegenerative diseases using rats with brain iron overload, recovering normal levels of hippocampal dynamin 1 like (DNM1L), caspase 3, and synaptophysin levels (da Silva et al. 2014). The DNM1L gene is responsible for encoding Dynamin‐1, a protein active in the process of mitochondrial fission that has its expression negatively affected in the studied model. In this sense, in addition to CBD returning the protein's basal expression levels, it also decreased the expression of caspase 3, which plays a role in apoptosis, and decreased the expression levels of synaptophysin, a protein that presents high levels of expression and is associated with low scores in tests that evaluate neuropsychological measures in patients with AD (Sze et al. 1997; da Silva et al. 2014; Lee et al. 2017).

#### 3.1.5 CBD Modulates the Cholinergic System

In ad, the cholinergic system comprises an important mechanism. In this pathway, there are two important cholinesterase enzymes, butyrylcholinesterase (BChE) and acetylcholinesterase (AChE); the latter is more expressed in the brain and regulates brain synapses by acetylcholine hydrolysis. Dysfunctions in the activity of these enzymes are commonly described in studies using ad models, such as a report of animals induced to develop a dementia‐like condition that resembles ad, through the cerebroventricular infusion of streptozotocin (Silva et al. 2023). The association between the disruption of the cholinergic system disturbance and ad has increased since significant symptomatic improvement has been observed in patients with AD treated with cholinesterase inhibitors (Summers et al. 1986). However, although still in use today, drugs that inhibit these enzymes have significant adverse reactions and only act to slow down the progression of the disease (Hampel et al. 2018; Han et al. 2019; Xu et al. 2021).

Given the necessity to formulate new treatment strategies, CBD has emerged as an interesting possibility not only for its neuroprotective effects, but also for its anticholinesterase properties. This hypothesis is supported by studies showing a decrease in AChE activity after treatment with CBD in cell culture, *C. elegans* , and Zebrafish models (Mooko et al. 2022; Vanin et al. 2022). In addition to proving the effect of CBD on AChE, the main cholinesterase enzyme related to the modulation of brain cholinergic synapses, treatments based on this cannabinoid also had an effect on decreasing BChE activity (Jiang et al. 2021; Patil et al. 2023).

#### 3.1.6 CBD Effects on Glucose and Lipid Metabolism

Other pathways related to AD include glucose and lipid metabolism. In Alzheimer's brains, positron emission tomography (PET) scans indicate low glucose metabolism. The effect of intraperitoneal CBD treatment on glucose metabolism in AD brains was first described in a study using PET imaging and rats induced to develop the disease by the use of streptozotocin, in which it was concluded that CBD maintains regular glucose metabolism. At the same time, untreated animals showed regions of hypometabolism near the striatum, motor cortex, hippocampus, and thalamus, further proposing the feasibility of CBD treatment as a possible method of early treatment for dementia (de Paula Faria et al. 2022).

In the context of lipid metabolism, insulin, an important regulator of lipid storage and processing in organisms, has an influence on the development of AD, since insulin resistance can lead to oxidative stress and inflammation (De Felice et al. 2014; Kandimalla et al. 2017). In vivo and in vitro studies indicate CBD as a reducing agent of cerebral insulin resistance, mainly by modulating the de novo synthesis of ceramides and acting on salvage pathways. In addition, results obtained in these studies reinforce the antioxidant role of CBD, since the cannabinoid decreased lipid peroxidation and has a protective effect through the decrease in tau protein phosphorylation (Charytoniuk et al. 2021; Vanin et al. 2022).

#### 3.1.7 CBD‐Induced Functional and Cognitive Improvements

In summary, CBD has numerous molecular interactions in important pathways related to AD. The result of this complex network of interactions can be seen as the basis for the broad physiological changes resulting from treatment with this compound. In humans, it was observed that treatment with CBD increased cerebral blood flow in key regions involved in processing memories, such as the hippocampus (Bloomfield et al. 2020). Another study, carried out on an in vitro model, indicated that pre‐treatment with CBD rescued the deficit caused by Aβ in long‐term potential (LTP), a phenomenon involved in signal transmission between two neurons, thereby stimulating them (Hughes and Herron 2019). LTP is one of the parameters related to synaptic plasticity and is believed to act in the encoding of memories in the brain, so it is considered the basis for learning and memory processes (Bliss and Collingridge 1993; Cooke 2006).

Regarding the changes in clinical aspects resulting from CBD administration, many reports address the drug's ability to improve conditions related to cognition and memory processing. Study models of ad, using rats and mice treated with CBD for different periods and with various concentrations, showed positive results in terms of memory processing and exploratory behavior, compared to control animals (Aso et al. 2014, 2016; Cheng, Low, et al. 2014; Cheng, Spiro, et al. 2014; Watt et al. 2020; Amini and Abdolmaleki 2022; Chesworth et al. 2022; Coles et al. 2022; de Paula Faria et al. 2022; Kreilaus et al. 2022). In addition to animal models, one research study evaluated the effect of a treatment based on the daily use of 3% CBD on human patients listed in the Alzheimer Hellas database with severe behavioral and physiological symptoms of dementia (BPSD). In this analysis, the CBD‐treated group showed a significant improvement in BPSD, compared to the conventional treatment group, making CBD a possible treatment method for these conditions as soon as it undergoes further randomized clinical trials (Alexandri et al. 2023).

**FIGURE 3:**
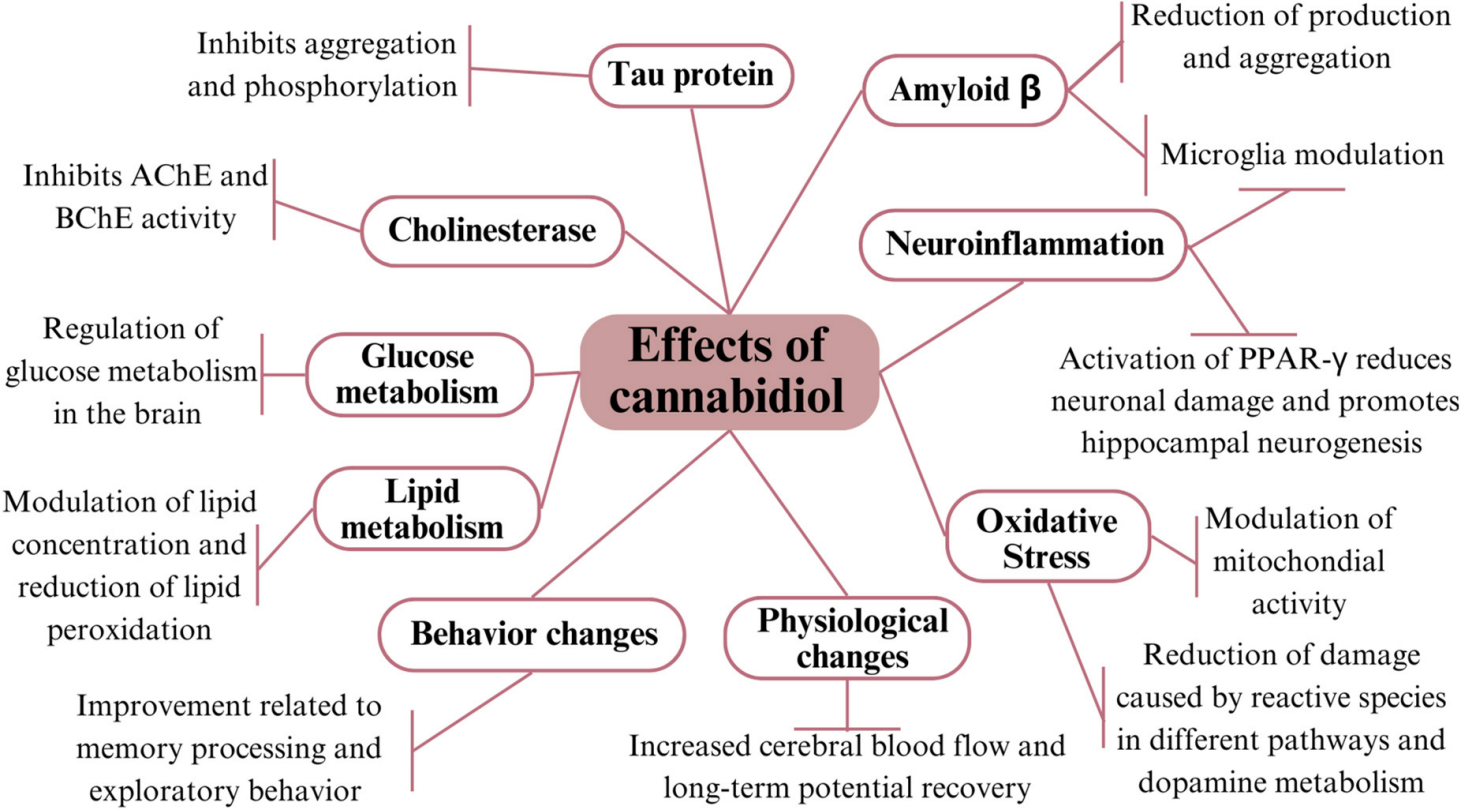
Mechanisms of action of cannabidiol in Alzheimer's disease. Schematic representation of the effects of CBD associated with AD found in the literature review.

Investment in studies aimed at understanding the diverse effects of CBD on aspects of ad has provided a wide range of information on the mechanisms related to this therapeutic approach. A synthesis of these findings can be seen in Figure 3, which demonstrates the multiple mechanisms of action of this compound, offering several possibilities for the investigation of new treatment methods based on CBD.

### 3.2 In Silico Identification of Key Therapeutic Pathways Modulated by CBD

**FIGURE 4:**
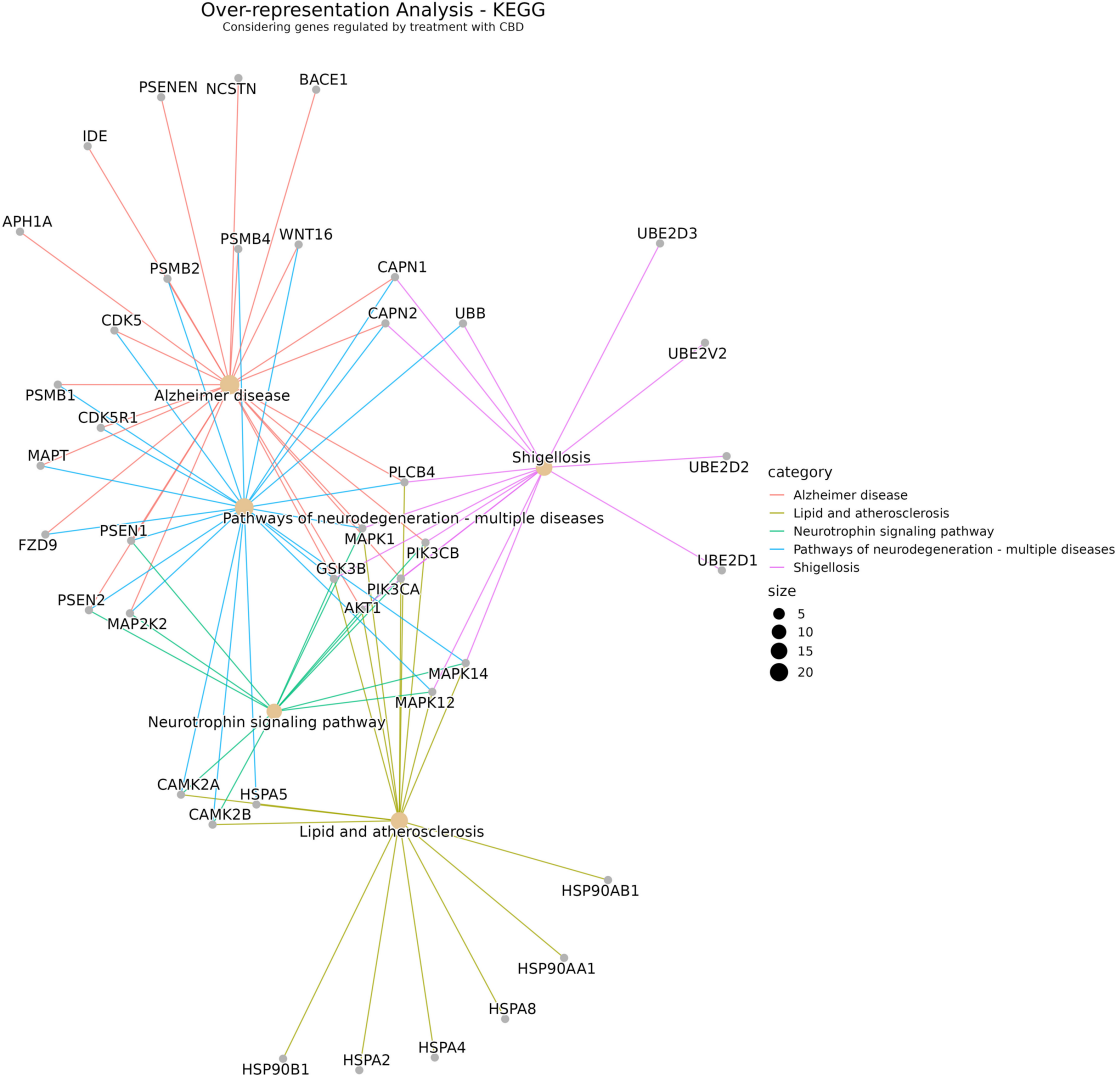
Enrichment network of KEGG pathways related to the Alzheimer disease. Obtained through the analysis of a set of 64 genes modulated by CBD treatment, obtained from the literature, in an Over‐Representation Analysis (ORA) using the clusterProfiler package in R and the KEGG gene set. The defined parameters were as follows: pvalueCutoff = 0.05, qvalueCutoff = 0.05, universe = org. Hs.eg.db, minGSSize = 10, maxGSSize = 500.

In ORA, five KEGG pathways were identified: AD (H00056 code in the database), lipid and atherosclerosis pathways (map 05417), neurotrophin signaling pathway (HSA04722), pathways of neurodegeneration—multiple diseases (HSA05022), and Shigellosis (HSA05131). The relationships between genes and pathways can be seen in Figure 4.

#### 3.2.1 CBD Modulates Key Genes in Aβ Formation and Neurodegeneration Pathways

The AD category is related to the mechanisms of pathology development, including genes and variants associated with processes of apoptosis, autophagy, impaired neurotransmission, mitochondrial abnormalities, impaired synaptic plasticity, and neurodegeneration. Some of these genes are associated with the production of Aβ, such as *PSEN1* and *PSEN2*, genes strongly associated with early‐onset ad (Do et al. 2023). These genes encode presenilins, proteins that constitute the γ‐secretase protein complex, which processes type I transmembrane proteins, such as the Aβ precursor protein (Hutton 1997; Fraser et al. 2000). Thus, as indicated in the work of Libro et al. (2016), the decrease in the expression of these genes and others related to the formation of the γ‐secretase complex—such as aph‐1 homologue A (*APH1A*), presenilin enhancer (*PSENEN*), nicastrin (*NCSTN*), as well as beta‐secretase 1 (*BACE1*), which encodes the β‐secretase enzyme also related to the production of Aβ—indicates that treatment with CBD may lead to a decrease in the formation of Aβ plaques and have a beneficial effect in the AD therapeutics (Fraser et al. 2000; Kitazume et al. 2001; Libro et al. 2016).

Genes of the AD pathway linked to the neurodegeneration pathway were identified as well. This category comprises processes that include abnormal protein dynamics due to factors such as deficiency in the ubiquitin‐proteasome‐autophagy system; oxidative stress and free radical formation; endoplasmic reticulum stress; mitochondrial dysfunction; and axonal transport interruptions. In addition, other gene families can be cited as a common point between these pathways, such as the class of protein subunits encoded by the PSMB genes. They take part in the formation of the proteasome complex, which has dysfunctions related to oxidative stress and the development of AD (Bonet‐Costa et al. 2016; Moya‐Alvarado et al. 2016).

Other genes modulated by CBD and related to both pathways are the calpain 1 (*CAPN1*) and 2 (*CAPN2*) genes, which encode calcium‐activated intracellular proteins whose increased activity is related to cognitive disorders, as in the case of AD, and the cyclin dependent kinase 5 (*CDK5*) and cyclin dependent kinase 5 regulatory subunit 1 (*CDK5R1*) genes (Saito et al. 1993; Ferreira and Bigio 2011). Unlike other genes in the cyclin dependent kinase (CDK) family, which are related to cell cycle regulatory proteins, the *CDK5* gene encodes a cyclin‐dependent protein kinase that is highly expressed in neurons of the CNS. Its main function is to act on synaptic plasticity and neuronal migration through the synaptic phosphorylation of proteins needed for the organization of the cytoskeleton, endocytosis, exocytosis, and apoptosis (Liu et al. 2016). A study investigating the modulation of CDK proteins in cases of AD shows increased expression of these proteins in the disease condition (Lim et al. 2021). Therefore, since CBD decreases the expression of these proteins, it may indicate a protective effect in the AD context.

#### 3.2.2 CBD's Impact on Lipid‐Related and Chaperone‐Mediated Pathways

The lipids and atherosclerosis category is characterized by processes related to lipid metabolism and atherosclerosis, a chronic inflammatory disease caused by the accumulation of lipid plaques on artery walls, leading to complications involved in cardiovascular diseases. Among the genes related to this pathway, some demonstrate links with a greater number of pathways and consequent cumulative action in ad, others present a punctual action. This is the case of genes from the CAMK2 family and genes related to the activity of heat shock proteins (HSPs). These proteins are part of a group of chaperones that regulate the folding, signaling, monitoring, and maintenance of cellular proteins, conferring a protective effect on the cells of the nervous system in cases of neuropathology (Macario and De Conway Macario 2007; Marino Gammazza et al. 2016). Studies have shown an important role for HSPs in inhibiting Aβ aggregation, in binding to ubiquitin, and in protein degradation, including that of Aβ and tau (Walls et al. 2012; Mateju et al. 2017; Meriin et al. 2018). Thus, the findings related to the upregulation of these genes with CBD treatment may indicate an interesting strategy to regulate pathways associated with ad development (Libro et al. 2016).

Moreover, the CAMK2 gene family was identified in this pathway, which includes some of the protein kinases related to the phosphorylation of tau protein in ad. In the context of atherosclerosis, CAMK2 members act in the regulation of inflammatory processes, since they promote the release of pro‐inflammatory proteins (Liu et al. 2008), which is a hallmark condition in the pathology of atherosclerosis. One of the induction pathways for inflammatory reactions occurs through the triggering of a metabolic cascade that results in the release of interleukins involving the activation of mitogen‐activated protein kinase (MAPK) (Zhang et al. 2011). Also named extracellular signal‐regulated kinases (ERKs), MAPKs play a fundamental role in the communication and integration of various biochemical signals within cells. Their action extends to a wide range of cellular processes, including proliferation, differentiation, transcription regulation, and development. After activation, MAPKs migrate to the cell nucleus, where they exert their function by phosphorylating various nuclear targets. This phosphorylation triggers metabolic cascades regulated by the action of these proteins (Blum and Dash 2009; Cargnello and Roux 2011). This family of proteins is encoded by numerous genes in which CBD has a modulating function, as well as playing a role in various pathways related to the development of ad. One such example is *MAP 2K2*, which is associated with the development of AD, pathways of neurodegeneration, and the neurotrophin signaling pathway (Aso et al. 2014).

#### 3.2.3 CBD Regulation of Neurotrophin, Ubiquitination, and Kinase Signaling Pathways

The neurotrophin signaling pathway, despite being related to the fewest genes compared to the others, was genes closely linked to at least two other pathways in the analysis carried out. This pathway includes mechanisms related to neurotrophins, a family of trophic factors involved in the differentiation and maintenance of neuronal cells. The regulation of these mechanisms is related to various regulatory cascades, which include signaling pathways mediated by kinases. Among the genes that represent the pathway are those encoding presenilins, mentioned previously, and proteins of the MAPK family, which are negatively modulated by treatments with CBD, as well as other related genes encoding other kinases such as phosphatidylinositol 3 (*PIK3C*) and human serine–threonine kinase (AKT) (Aso et al. 2014; Libro et al. 2016).

In addition to MAP 2K2, other genes from the MAPK family are modulated by the effect of CBD and are related to ad. The *MAPK12* and *MAPK14* genes have been linked to pathways involved in neurodegeneration, neurotrophin signaling, lipids, and atherosclerosis and the pathway related to Shigellosis. Shigellosis, or bacillary dysentery, is an intestinal infection caused by Shigella, a genus of enterobacteria. Shigella are potential food‐borne pathogens that are able to colonize the intestinal epithelium, exploiting the functions of epithelial cells and bypassing the host's innate immune response (Echeverria et al. 1991). The enrichment of this pathway in our functional analysis was not expected in the context of ad. This finding could be explained by the effect of CBD on genes related to ubiquitination processes, which correspond to some of the genes associated with the Shigellosis pathway. Ubiquitination consists of a post‐transcriptional modification mediated by proteins that insert an ubiquitin into a target protein, which leads to different processes, such as protein degradation, subcellular localization, and kinase activation (Callis 2014). The ubiquitination mechanism is crucial in the process of Shigella infection, as the bacterium exploits the host's ubiquitin‐proteasome system via bacterial ubiquitin ligases. This facilitates the targeted degradation of proteins involved in the host's immune defense, thereby aiding in the establishment and progression of the infection (Tanner et al. 2015; Wandel et al. 2017).

Since ad involves the abnormal accumulation of Aβ proteins and a large part of the protein clearance mechanisms depends on ubiquitination, this pathway plays an important role (Glickman and Ciechanover 2002; McKinnon and Tabrizi 2014; Tramutola et al. 2016; Zhang et al. 2017). Based on this mechanism, a study proposes a three‐dimensional model for studying ad in human neural cell cultures induced by alterations in the ubiquitin signaling pathway (Maniv et al. 2023). In this case, treatments that result in the positive modulation of genes related to the ubiquitin signaling pathway, like CBD, may reduce the protein accumulation in affected brains.

The regulation of signaling hubs seems to be a good target for understanding and treating the pathology involved in ad and CBD has shown promising effects in this regard. Regulatory mechanisms associated with the action of neurotrophins, ubiquitins, and signaling related to protein kinases were studied. This must be highlighted since the genes related to these mechanisms interact with all the addressed pathways found in the analysis. As an example, we have the *MAPK1* gene, which plays a role in all the pathways shown in Figure 2.

The *PIK3CA* and *PIK3CB* genes are related to the expression of the PIK3 protein. The activity of this protein, together with the action of the protein kinase 3 (AKT), one of whose related genes is *AKT1*, participates in the important PIK3/AKT signaling axis with a wide range of functions in the brain, regulating cell survival, proliferation, growth, differentiation, motility, intracellular traffic, and the extension of neurites (dendrites and axons) (Vanhaesebroeck et al. 2010; Ye et al. 2019). In the AD brain, accumulated Aβ prevents the propagation of the PIK3/Akt signaling axis and increases the activity of this protein kinase in neurons, eliminating the suppressive effect of this pathway on GSK‐3. In addition to the indirect impact on GSK‐3, Aβ oligomers could also directly stimulate this kinase in neurons and neuron stem cells. In turn, activated GSK‐3 could increase apoptotic signals and decrease cell survival capacity. This enzyme also plays a profound role in regulating tau hyperphosphorylation. In fact, activation of the PI3K/Akt signaling axis in neurons could reduce tau hyperphosphorylation by suppressing GSK‐3 activity (Ariga et al. 2008; Choi and Ho Koh 2016; Ryu et al. 2016; Ren et al. 2018). In addition to this mechanism, the PIK3/Akt signaling axis plays a regulatory role in pathways related to glucose and insulin metabolism, changes in autophagy processes, oxidative stress, and neuroinflammation. A review by Razani et al. (2021) provides a robust and detailed summary of the molecular mechanisms that drive the effects of this pathway in AD brains (Razani et al. 2021).

Even though more studies are needed to reveal CBD's modulating effect at a systemic level, therapeutics based on CBD show interesting relevance due to its potential to modulate genes related to important signaling pathways, such as those of the PIK3 family, *AKT1*, and *GSK3β* (Aso et al. 2014; Libro et al. 2016).

In silico analysis identified important pathways involved in ad and related mechanisms, such as neurodegeneration, inflammatory processes, and neurotrophic signaling. The genes regulated by CBD treatment are related to pathological mechanisms, such as the accumulation of Aβ plaques, mitochondrial dysfunction, and oxidative stress, reinforcing the beneficial effect of this cannabinoid at the molecular level. In addition, CBD treatment demonstrated promising effects by regulating the expression of crucial genes, such as those linked to the γ‐secretase complex and protein kinase signaling, suggesting a possible reduction in Aβ plaque formation and tau phosphorylation. Thus, CBD may play a protective role by regulating pathways such as ubiquitination, PIK3/AKT signaling, and lipid metabolism, reinforcing its therapeutic potential for ad. Although further studies are needed, CBD presents itself as a relevant therapeutic alternative due to its ability to genetically modulate key ad processes.

### 3.3 CBD as a Complementary Method for the Treatment of ad

With the data presented in this study, the beneficial effects of CBD on key pathological pathways of ad are evident. However, when considering the implementation of a new therapeutic approach, special attention should be paid to adverse effects, optimal dosing strategies, and long‐term safety in clinical settings.

In this sense, despite promising preclinical findings, current clinical evidence for CBD remains limited. Most studies to date have used heterogeneous formulations and small sample sizes, often combining CBD with other cannabinoids such as THC, which makes interpretation of results difficult (Singh et al. 2023; Martiniano et al. 2024). The lack of randomized phase III clinical trials specifically focused on ad represents a significant barrier to clinical translation.

In addition, although CBD is generally considered well tolerated, safety concerns have been raised, particularly at high doses or in combination with other medications. A meta‐analysis demonstrated a dose‐dependent increase in the risk of elevated liver enzymes and drug‐induced liver injury (Lo et al. 2023). Furthermore, CBD inhibits several cytochrome P450 enzymes, increasing the potential for significant pharmacokinetic interactions, especially in elderly patients on polypharmacy regimens (Huestis et al. 2019).

In contrast, conventional therapies such as acetylcholinesterase inhibitors are supported by a solid clinical evidence base. Studies have confirmed their modest but consistent cognitive benefits (Ritchie 2004; Moreta et al. 2021). However, they are often associated with adverse effects, particularly gastrointestinal and cardiovascular complications, including bradycardia, atrioventricular block, and increased risk of hospitalization, especially in the first months of use (Gauthier 2001; Ruangritchankul et al. 2021). In this context, CBD may be better positioned as a complementary therapy rather than a replacement for established treatments. Its multimodal mechanisms—anti‐inflammatory, antioxidant, and receptor modulation—combined with a favorable safety profile at therapeutic doses, suggest its potential for integration into combination regimens (Fernández‐Ruiz et al. 2013; Noreen et al. 2018). This may be particularly beneficial for patients who are intolerant to cholinesterase inhibitors or who have neuropsychiatric symptoms not well controlled by current medications. To expand this potential, more clinical research is needed to define optimal dosing regimens, evaluate long‐term outcomes, and establish safety parameters in elderly and comorbid populations. Regulatory challenges and standardization of formulations must also be addressed to enable safe and effective clinical use of CBD in ad.

## 4 Conclusion

To the authors' knowledge, this is the first work to collectively address the key findings about the role of CBD in ad, adding in silico enrichment analysis to provide a comprehensive overview of the genes whose expression levels are modulated by CBD treatment.

The bibliographic analysis presented a rich understanding of CBD's action mechanisms. The results point to a broad action of CBD, with effects that can be positive in several aspects of ad, such as reducing the production and aggregation of amyloid beta protein, as well as decreasing the hyperphosphorylation of tau protein. CBD is also capable of decreasing the effect of AChE and BChE, a mechanism used to decrease the progression of the disease through other anticholinesterases, as well as regulating processes associated with glucose and lipid metabolism, also decreasing the tissue inflammatory response and oxidative stress. The combination of these effects related to CBD treatment may explain the behavioral improvement observed in in vivo studies and the beneficial physiological changes reported in the literature. Therefore, even though most of the explored studies are in a pre‐clinical scope, these data support a promising future regarding CBD‐based treatments for patients with AD.

The in silico analysis performed corroborates the idea of the positive effect of CBD for the treatment of ad. The identification of key ad pathways, such as inflammatory processes, signaling mechanisms, processing of Aβ and tau proteins, and ubiquitination pathways based on genes regulated by the treatment, provides a greater understanding of the molecular bases that act on the therapeutic effect of CBD observed in preclinical studies. Furthermore, this analysis identified genes that are central to ad‐associated pathways. These genes are of great interest since they may act as targets for the regulation of several mechanisms that promote ad‐associated dementia and may also experience a cumulative positive effect of CBD treatment, as they are involved in multiple pathways influenced by it. In this sense, great effort is recommended to analyze the mechanisms and effects related to their modulation.

As limitations, the literature review aimed to survey the results of CBD treatments already associated with ad; thus, other studies that demonstrate the CBD effects on mechanisms associated but without directly relating it to the disease were left out. Additionally, no studies were identified that examine the potential adverse effects or toxicity associated with CBD treatments in the search performed.

Another limitation is the limited amount of data on the effect of CBD on the expression of genes associated with ad. Studies involving candidate genes were not used for this analysis, in order to avoid bias in the identification of the main affected pathways. Thus, this analysis can be further enriched as more studies evaluating gene expression after CBD treatment are performed. In this sense, research based on hypothesis‐free analysis methodologies that provide a broader view of the effect of CBD on ad is encouraged.

In summary, the combination of literature review and in silico analysis brings together classical and contemporary data analysis methods, promoting a rich understanding of a complex disease such as ad. This combination of methodologies, capable of bringing together the effects and pathways of action associated with genetic modulation promoted by CBD treatment, demonstrates the enabling potential of this cannabinoid for the development of a complementary therapeutic method for ad.

## Author Contributions

**João V. Mello‐Hortega:** conceptualization, investigation, methodology, writing – original draft. **Carolina S. de Oliveira:** writing – review and editing. **Vitoria S. de Araujo:** software. **Lupe Furtado‐Alle:** writing – review and editing. **Luciane V. Tureck:** funding acquisition, writing – review and editing. **Ricardo L. R. Souza:** conceptualization, project administration, supervision, writing – review and editing.

## Conflicts of Interest

The authors declare the following financial interests/personal relationships which may be considered as potential competing interests: Joao V M Hortega reports financial support was provided by Coordenação de Aperfeiçoamento de Pessoal de Nível Superior; Carolina S. de Oliveira reports financial support was provided by Conselho Nacional de Desenvolvimento Científico e Tecnológico.

## Peer Review

The peer review history for this article is available at https://www.webofscience.com/api/gateway/wos/peer‐review/10.1111/ejn.70229.

## Supporting information


**Table S1:** Supporting Information.

**Table S2:** Supporting Information.
