# Supplementary material for: Cannabidiol and Alzheimer Disease: A Comprehensive Review and In Silico Insights Into Molecular Interactions
Source: Eur J Neurosci. 2025 Aug 27;62(4):e70229. doi: 10.1111/ejn.70229 (PMC12381694; doi:10.1111/ejn.70229)
Supplement: Supplementary file 1 — Table S1: Supporting Information. [file EJN-62-0-s002.docx]

| Pathway affected by cannabidiol | Development mechanism affected by Cannabidiol | Cannabidiol effect | Study model | Reference |
| --- | --- | --- | --- | --- |
| Amyloid-β pathway | Amyloid-β accumulation  Amyloid-β production | CBD coated by nano-chitosan has good potential for reducing Aβ plaques, increasing CB1 and CB2 levels. | Rats that receive amyloid beta peptide. | (Amini & Abdolmaleki, 2022a) |
|  |  | CBD scavenges reactive oxygen species alleviating the aggregation of Aβ. | *C. Elegans* model. | (Zhang *et al.*, 2022) |
|  |  | CBD is able to decrease the aggregation of peptides at certain concentrations of compounds in water. | *In silico* | (Chrobak *et al.*, 2021) |
|  |  | Moderate brain region-specific reduction in soluble Aβ levels | Male APPxPS1 mice. | (Aso *et al.*, 2014; Watt *et al.*, 2020) |
|  |  | Extracts with CBD reduced the deposition in the hippocampus and cerebral cortex | (PK-/-/TAUVLW) mice model. | (Casarejos *et al.*, 2013) |
|  |  | Regulation of theWnt/Β-catenin pathway and PPARγ, decreasing the processing of Aβ precursor. | PC12 cells. | (Vallée *et al.*, 2017) |
|  | Amyloid-β production | CBD led to the downregulation of genes coding for the secretases involved in Aβ generation. | Mesenchymal stem cells. | (Libro *et al.*, 2016) |
|  |  | Ubiquitination of APP protein with the consequent decrease in Aβ production. | SH-SY5YAPP+ cells. | (Scuderi *et al.*, 2014) |
|  |  | Acts as an inverse agonist to inhibit β-arrestin 2 recruitment to GPR3, reducing Aβ generation. | Chinese hamster ovary (CHO)-K1 cells coexpressing EA-β-arrestin2. | (Woelfl *et al.*, 2020) |
|  |  | Extracts with CBD showed inhibitory potential towards β-secretase enzyme activity. | Pre-adipocytes (3T3-L1) (CL-173). | (Aso *et al.*, 2014) |
|  | Microglia Activation | In synergy with β-asarone, it downregulates apoptosis-associated genes and promotes autophagy caused by Aβ. | CL4176 *C.elegans*  model. | (Duan *et al.*, 2023) |
|  |  | Enhanced microglial Aβ phagocytosis through TRPV2 activation. | Human dataset, mouse primary neuron and microglia cultures, and AD model mice. | (Yang *et al.*, 2022) |
|  |  | Inhibit induced Aβ-neurotoxicity | PC12 cells. | (Marsh *et al.*, 2023) |
|  |  | Promotes immune system response and autophagy pathway up-regulation | APP/PS1 mice. | (Hao & Feng, 2021) |
| Tau pathway | Tau aggregation | Through the spontaneous binding of the cannabidiol molecule with the tau molecule, it affects the protein’s LAG phase, suppressing tau fibrils’ aggregation. | *Escherichia coli* BL21 strain DE3. | (Alali *et al.*, 2021) |
|  |  | Extracts with CBD reduced the deposition in the hippocampus and cerebral cortex | (PK-/-/TAUVLW) mice model. | (Casarejos *et al.*, 2013) |
|  | Tau phosphorylation | Regulation of the Wnt/Β-catenin pathway and PPARγ, reducing tau phosphorylation. | PC12 cells. | (Cristino *et al.*, 2020) |
|  |  | CBD led to the downregulation of genes coding for the kinases responsible for tau phosphorylation. | Mesenchymal stem cells. | (Libro *et al.*, 2016) |
|  |  | Reduction of tau phosphorylation mediated by Wnt pathway. | PC12 cells. | (Esposito, De Filippis, Carnuccio, *et al.*, 2006) |
| Neuroinflammation | Microglia activation | Blocking the activation of microglia, preventing the transition from M0 to M1 phenotype. | Rat organotypic slices exposed to kainate, in vitro seizure model. | (Landucci *et al.*, 2022) |
|  |  | Modulate of microglial cell function and prevent cytokine gene expression. | N13 microglial cells and Amyloid-β-injected mice. | (Martín-Moreno *et al.*, 2011) |
|  |  | Upregulates IL-33 and TREM2 genes | 5XFAD mice. | (Khodadadi *et al.*, 2021) |
|  |  | CBD increased the expression of FAAH and CB1. | *C. Elegans* and SH-SY5Y cells. | (Wang *et al.*, 2021) |
|  |  | Regulates GFAP mRNA and protein expression, and impairs INOS and IL-β protein expression. | Aβ-injected mice. | (Esposito *et al.*, 2007) |
|  |  | Inhibits nitrite production and INOS protein expression. | PC12 cells. | (Esposito, De Filippis, Maiuri, *et al.*, 2006) |
|  | PPARγ modulation | Activation of PPARγ reduces neuronal damage and promotes hippocampal neurogenesis. | Rat AD model. | (Esposito *et al.*, 2011) |
| Oxidative stress | Glyoxalase pathway | Prevention of cell damage mediated by methylglyoxal through enhancement of the neural glioxalase pathway. | C. Elegans strain modeling Alzheimer's disease. | (Frandsen & Narayanasamy, 2022) |
|  | Wnt/Β-catenin pathway and PPARγ | Regulation of the pathway, reducing oxidative stress. | PC12 cells. | (Vallée *et al.*, 2017) |
|  | Reactive oxygens species | CBD was found to scavenge reactive oxygen species without inducing the overexpression of oxidative genes. | C. Elegans model. | (Zhang *et al.*, 2022) |
|  |  | Extracts with CBD reduces ROS levels | C. Elegans model. | (Vanin *et al.*, 2022; Wang *et al.*, 2023) |
|  |  |  | PC12 cells. | (Iuvone *et al.*, 2004) |
|  | Mitochondrial dynamics | CBD rescued iron-induced effects, bringing hippocampal DNM1L, caspase 3 and synaptophysin levels back. | Rats with brain iron overload. | (da Silva *et al.*, 2014) |
|  |  |  | PC12 cells. | (Iuvone *et al.*, 2004) |
|  |  | Modulates expression of genes related to the mitochondrial function. | HGMSCS cells. | (Rajan *et al.*, 2017) |
|  | Axis NRF2-BACH1 | Inhibits BACH1. | Cell lineages. | (Casares *et al.*, 2020) |
|  | Dopamine metabolism | Extracts with CBD reduce free radical production in the pathway. | (PK-/-/TAUVLW) mice model. | (Casarejos *et al.*, 2013) |
|  | - | Improved cell viability in response to Tert-Butyl Hydroperoxide. | PC12 and SH-SY5Y cells. | (Harvey *et al.*, 2012) |
| Cholinergic pathway | Acetylcholinesterase activity | Extracts with CBD showed inhibitory potential towards cholinesterase activity. | Pre-adipocytes (3T3-L1) (CL-173). | (Mooko *et al.*, 2022) |
|  |  |  | Zebrafish model. | (Vanin *et al.*, 2022) |
|  |  |  | *C. Elegans* model. | (Vanin *et al.*, 2022) |
|  | Butyrylcholinesterase activity | Extracts with CBD showed inhibitory potential towards cholinesterase activity. | Pre-adipocytes (3T3-L1) (CL-173). | (Mooko *et al.*, 2022) |
|  |  | Has the potential to inhibit BChE. | PC12 cells and ICR mice. | (Jiang *et al.*, 2021) |
|  |  | Inhibits BChE activity. | In silico. | (Patil *et al.*, 2023) |
| Glucose metabolism | Glucose hypometabolism (characteristic of a brain with Alzheimer's disease) | Regulation of glucose metabolism in the brain. | Strptozotocin-induced Alzheimer's disease rat model. | (de Paula Faria *et al.*, 2022) |
| Lipid metabolism | De novo synthesis and salvage pathway | Modulates the concentration of sphingolipids, ceramide and sphingomyelin | Rats that received a high-fat diet. | (Charytoniuk *et al.*, 2021) |
|  |  | Decreased lipid peroxidation | PC12 cells. | (Vanin *et al.*, 2022) |
| Behavioral changes | Memory processing | Protects/Improves pathways related to memory processing and storage related to spacial and social recognition. | Strptozotocin-induced Alzheimer's disease rat model. | (de Paula Faria *et al.*, 2022) |
|  |  |  | Rats that receive amyloid beta peptide. | (Amini & Abdolmaleki, 2022b) |
|  |  |  | APPxPS1 mice. | (Aso *et al.*, 2014, 2016; Cheng *et al.*, 2014, 2014; Watt *et al.*, 2020; Coles *et al.*, 2022) |
|  |  |  | Female Tau58/2 mice. | (Kreilaus *et al.*, 2022) |
|  | Exploratory behavior | Reduces latency in finding a food reward and increases locomotion. | Female APPxPS1 mice. | (Chesworth *et al.*, 2022) |
|  | Behavior and Physical Symptoms in Dementia (BPSD) | Improvement of the BPSD in patients. | AD patients. | (Alexandri *et al.*, 2023) |
| Physiological changes | Cerebral blood flow (CBF) | Increases CBF to key regions involved in memory processing, particularly the hippocampus. | An arterial spin labeling magnetic resonance imaging study in humans. | (Bloomfield *et al.*, 2020) |
|  | Long-term potential (LTP) | Pre-treatment rescued the Aβ mediated deficit in LTP. | *In vitro* model of AD. | (Hughes & Herron, 2019) |
