# Supplementary material for: Cannabidiol and Alzheimer Disease: A Comprehensive Review and In Silico Insights Into Molecular Interactions
Source: Eur J Neurosci. 2025 Aug 27;62(4):e70229. doi: 10.1111/ejn.70229 (PMC12381694; doi:10.1111/ejn.70229)
Supplement: Supplementary file 2 — Table S2: Supporting Information. [file EJN-62-0-s001.docx]

| GENES | REGULATION | SAMPLE | REFERENCE |
| --- | --- | --- | --- |
| CAMK2A | Downregulated | Gingiva Mesenchymal stem cells (MSCS) | (Libro *et al.*, 2016) |
| CAMK2B | Downregulated | Gingiva Mesenchymal stem cells (MSCS) |  |
| CAPN1 | Downregulated | Gingiva Mesenchymal stem cells (MSCS) |  |
| CAPN2 | Downregulated | Gingiva Mesenchymal stem cells (MSCS) |  |
| CDK5 | Downregulated | Gingiva Mesenchymal stem cells (MSCS) |  |
| CDK5R1 | Downregulated | Gingiva Mesenchymal stem cells (MSCS) |  |
| DYRK1A | Downregulated | Gingiva Mesenchymal stem cells (MSCS) |  |
| GSK3B | Downregulated | Gingiva Mesenchymal stem cells (MSCS) |  |
| MAPK1 | Downregulated | Gingiva Mesenchymal stem cells (MSCS) |  |
| MAPK12 | Downregulated | Gingiva Mesenchymal stem cells (MSCS) |  |
| MAPK14 | Downregulated | Gingiva Mesenchymal stem cells (MSCS) |  |
| MAPT | Downregulated | Gingiva Mesenchymal stem cells (MSCS) |  |
| APH1A | Downregulated | Gingiva Mesenchymal stem cells (MSCS) |  |
| BACE1 | Downregulated | Gingiva Mesenchymal stem cells (MSCS) |  |
| NCSTN | Downregulated | Gingiva Mesenchymal stem cells (MSCS) |  |
| PSENEN | Downregulated | Gingiva Mesenchymal stem cells (MSCS) |  |
| PSEN1 | Downregulated | Gingiva Mesenchymal stem cells (MSCS) |  |
| PSEN2 | Downregulated | Gingiva Mesenchymal stem cells (MSCS) |  |
| HSPA2 | Upregulated | Gingiva Mesenchymal stem cells (MSCS) |  |
| HSPA4 | Upregulated | Gingiva Mesenchymal stem cells (MSCS) |  |
| HSPA5 | Upregulated | Gingiva Mesenchymal stem cells (MSCS) |  |
| HSPA8 | Upregulated | Gingiva Mesenchymal stem cells (MSCS) |  |
| HSP90AA1 | Upregulated | Gingiva Mesenchymal stem cells (MSCS) |  |
| HSP90AB1 | Upregulated | Gingiva Mesenchymal stem cells (MSCS) |  |
| HSP90B1 | Upregulated | Gingiva Mesenchymal stem cells (MSCS) |  |
| UBB | Upregulated | Gingiva Mesenchymal stem cells (MSCS) |  |
| UBE2A | Upregulated | Gingiva Mesenchymal stem cells (MSCS) | (Libro *et al.*, 2016) |
| UBE2B | Upregulated | Gingiva Mesenchymal stem cells (MSCS) |  |
| UBE2D1 | Upregulated | Gingiva Mesenchymal stem cells (MSCS) |  |
| UBE2D2 | Upregulated | Gingiva Mesenchymal stem cells (MSCS) |  |
| UBE2D3 | Upregulated | Gingiva Mesenchymal stem cells (MSCS) |  |
| UBE2E1 | Upregulated | Gingiva Mesenchymal stem cells (MSCS) |  |
| UBE2E2 | Upregulated | Gingiva Mesenchymal stem cells (MSCS) |  |
| UBE2V2 | Upregulated | Gingiva Mesenchymal stem cells (MSCS) |  |
| UBE3A | Upregulated | Gingiva Mesenchymal stem cells (MSCS) |  |
| ACE1 | Upregulated | Gingiva Mesenchymal stem cells (MSCS) |  |
| ECE1 | Upregulated | Gingiva Mesenchymal stem cells (MSCS) |  |
| IDE | Upregulated | Gingiva Mesenchymal stem cells (MSCS) |  |
| ADAM9 | Upregulated | Gingiva Mesenchymal stem cells (MSCS) |  |
| PIK3CA | Upregulated | Gingiva Mesenchymal stem cells (MSCS) |  |
| PIK3CB | Upregulated | Gingiva Mesenchymal stem cells (MSCS) |  |
| AKT1 | Upregulated | Gingiva Mesenchymal stem cells (MSCS) |  |
| TMED10 | Upregulated | Gingiva Mesenchymal stem cells (MSCS) |  |
| ADCY3 |  | Hippocampus AβPP/PS1 Mice | (Aso *et al.*, 2014) |
| KIT |  | Hippocampus AβPP/PS1 Mice |  |
| PLCB4 |  | Hippocampus AβPP/PS1 Mice |  |
| MAPK2K2 |  | Hippocampus AβPP/PS1 Mice |  |
| FZD9 |  | Hippocampus AβPP/PS1 Mice |  |
| WNT16 |  | Hippocampus AβPP/PS1 Mice |  |
| GEMIN2 |  | Hippocampus AβPP/PS1 Mice |  |
| EIF3F |  | Hippocampus AβPP/PS1 Mice |  |
| SRRM1 |  | Hippocampus AβPP/PS1 Mice |  |
| POP1 |  | Hippocampus AβPP/PS1 Mice |  |
| GEMIN8 |  | Hippocampus AβPP/PS1 Mice |  |
| NUP35 |  | Hippocampus AβPP/PS1 Mice |  |
| PSMB1 |  | Hippocampus AβPP/PS1 Mice |  |
| PSMB2 |  | Hippocampus AβPP/PS1 Mice |  |
| PSMB4 |  | Hippocampus AβPP/PS1 Mice |  |
| CB1 |  | Hippocampus AβPP/PS1 Mice |  |
| CEBPA |  | Hippocampus AβPP/PS1 Mice |  |
| FGF2 |  | Hippocampus AβPP/PS1 Mice |  |
| KIT |  | Hippocampus AβPP/PS1 Mice |  |
| MAP2K2 |  | Hippocampus AβPP/PS1 Mice | [31] |
